# Fading the spots: a pilot case series of partial unilateral lentiginosis treated with Q-switched Nd: YAG laser

**DOI:** 10.1080/07853890.2026.2690719

**Published:** 2026-06-25

**Authors:** Alok Jindal, Kritika Bansal, Aditi Wadhwa, Shitij Goel

**Affiliations:** Department of Dermatology Venereology and Leprosy, Sharda University School of Medical Sciences and Research, Greater Noida, India

**Keywords:** Lentiginosis, pigmentation disorders, lasers, Nd:YAG lasers, dermoscopy, laser therapy

## Abstract

**Introduction:**

Partial unilateral lentiginosis (PUL) is rare pigmentary disorder characterized by clusters of unilateral lentiginous macules that can cause significant cosmetic and psychosocial distress. Despite its benign nature, there is no established standard treatment, and existing options often yield inconsistent results. This pilot case series evaluated the efficacy and safety of the Q-switched Nd:YAG laser (532 nm) in patients with PUL. About the Case Series.

**About the Case Series:**

Six patients (four females, two males; age range 10–32 years) underwent treatment with Q-switched Nd:YAG laser using the 532 nm wavelength. Given the rarity of the condition, this exploratory study was designed to descriptively evaluate clinical and dermoscopic outcomes rather than perform inferential statistical analysis. Treatment was initiated at low fluences and gradually titrated to a maximum of 2.5 J/cm² and 200 mJ, with sessions performed at 2–3 week intervals (mean of 23 sessions). Outcomes were assessed clinically, dermoscopically, and through patient-reported satisfaction, and were independently reviewed by three dermatologists. One patient achieved complete clearance, while the remaining five showed more than 70% improvement, with a mean dermoscopic pigment score reduction of approximately 68.6%. Patient satisfaction was high (mean VAS 8.8/10), with particular improvement noted in self-confidence and social interactions. Adverse effects were mild and included transient erythema and pigmentary changes, none of which required discontinuation.

**Conclusion:**

Q-switched Nd:YAG laser represents a safe, effective, and reproducible therapeutic option for PUL, offering substantial pigment clearance and meaningful improvement in patient quality of life.

## Introduction

Partial unilateral lentiginosis (PUL) is a rare pigmentary disorder characterized by clusters of discrete, hyperpigmented macules arising on otherwise normal skin. These lesions typically follow a segmental or checkerboard-like distribution and are confined to one side of the body, reflecting a form of cutaneous mosaicism. The condition was first described by McKelway in 1904 in a patient with unilateral involvement. Most cases present at birth or during early childhood, although adult-onset variants have also been reported [1,2].

While PUL is benign, the conspicuous distribution of lesions can lead to significant cosmetic and psychosocial concerns, particularly when the face or other exposed areas are involved. Diagnosis is primarily clinical, supported by dermoscopic findings, while histopathology in selected cases can help confirm the diagnosis by demonstrating increased basal layer pigmentation due to increased number of melanocytes, without melanocytic nesting, helping to differentiate PUL from other segmental pigmentary disorders such as nevus spilus or café-au-lait macules [3,4].

There is currently no standardized treatment for PUL. Conventional options such as chemical peels and non-specific laser techniques have shown inconsistent efficacy and carry risks of dyspigmentation or scarring. Pigment-specific lasers, particularly the Q-switched Nd:YAG, have demonstrated favorable outcomes in lentigines and other acquired pigmentary disorders, However, evidence specific to PUL remains limited and is largely confined to small case series, predominantly using the 1064-nm wavelength [5,6].

In recent years, dermatologic laser therapy has expanded beyond pigment removal alone, with increasing interest in laser-assisted delivery and regenerative approaches. While such strategies represent an evolving therapeutic frontier, their role in segmental pigmentary disorders such as PUL remains uncertain. Consequently, pigment-selective laser modalities continue to form the cornerstone of treatment for this condition [7,8].

This pilot case series aimed to evaluate the efficacy and safety of Q-switched Nd:YAG (532 nm) laser in patients with PUL using clinical, dermoscopic, and patient-reported outcome measures. Given the rarity of the condition and lack of standardized treatment protocols, the objective was to generate structured exploratory evidence rather than statistical inference.

## Methods

### Study design and sample size

This study was designed as a pilot case series to explore feasibility, safety, and clinical response of Q-switched Nd:YAG (532 nm) laser in PUL, a rare pigmentary disorder. Given the exploratory nature of the study and the low prevalence of the condition, the sample size was determined by feasibility rather than formal statistical calculation. No inferential statistical analysis was planned or performed, as the study was descriptive in nature and aimed to generate preliminary clinical and dermoscopic evidence.

### Laser treatment protocol

Written informed consent was obtained from all patients (and from guardians in the case of minors) before initiating the Laser treatments. All treatments were performed using a Q-switched Nd:YAG laser at 532 nm. Fluence was initiated at 0.2 J/cm^2^ and gradually titrated up to 2.5 J/cm^2^, with spot sizes ranging from 3–5 mm and pulse energies between 40–200mJ. Parameters were adjusted session-wise until the clinical endpoint of immediate whitening and transient erythema was achieved, without blistering or crusting. Sessions were scheduled at intervals of 2–3 weeks.

### Outcome assessment

Outcomes were assessed using multiple modalities:

- **Clinical assessment:** Standardized photographs were taken at baseline and final follow-up. Improvement was graded using the Quartile Grading Scale (QGS; 0–4) and Physician’s Global Assessment (PGA; 0–4).
- **Dermoscopy:** Digital dermoscopic images were obtained at baseline and after treatment. A dermoscopic pigment score (0–12) was used, summing four parameters (network density, color intensity, border definition, perifollicular clearing; each graded 0–3).
- **Patient-reported outcomes:** Patient satisfaction was evaluated using a Visual Analogue Scale (VAS; 0–10).
- **Safety:** Adverse events such as erythema, hypopigmentation, or scarring were documented at each visit.

All outcome assessments were independently reviewed and confirmed by three dermatologists with expertise in pigmentary disorders, ensuring consistency and minimizing observer bias.

### Follow-up

Patients were followed for an average of 6 months after the final session to assess durability of response and relapse.

## Results

The findings of this study are presented in terms of patient demographics, treatment parameters, and both dermoscopic and clinical outcomes. Treatment details, including laser fluence, pulse energy, and spot size, are described below, followed by dermoscopic and clinical responses (Table 2).

**Table 2.** Laser parameters and treatment outcomes.

Patient	Sessions	QGS	PGA	VAS	Final pigment score	Percentage improvement	Adverse events
1	26	3	3	8	4	70%	post inflammatory hypopigmentation, post inflammatory hyperpigmentation
2	24	4	4	10	0	100%	None
3	23	4	3	9	3	80%	None
4	22	4	3	9	3	80%	post inflammatory hypopigmentation
5	15	3	3	8	4	70%	post inflammatory hypopigmentation
6	28	4	3	9	3	75%	None

Note: QGS = Quartile Grading Scale; PGA = Physician Global Assessment; VAS = Visual Analogue Scale.

### Patient demographic and clinical profile

Patient demographics and clinical features are summarized in Table 1.

**Table 1.** Clinical and demographic characteristics of patients with partial unilateral lentiginosis.

Patient	Age/Sex	Skin type	Site	Duration (yrs)	Baseline pigment score
1	22/F	4	Chest, Neck	20	10
2	35/F	5	Face, Chest, Abdomen, Neck, Upper Limb	7	9
3	36/F	4	Upper Back, Neck	31	8
4	31/F	4	Chest	20	9
5	12/M	4	Face, Neck	11	9
6	38/M	4	Upper Back, Neck	32	9

### Laser parameters

Treatment was initiated with low fluence settings (∼0.2–0.5J/cm^2^) and pulse energies around 40mJ. Parameters were progressively increased session by session, depending on patient tolerance and lesion response. By later sessions, fluence was raised to a maximum of 2.5 J/cm^2^ and pulse energy up to 200mJ, while maintaining spot sizes between 3 and 5 mm. The clinical endpoint of immediate whitening and transient erythema was consistently achieved without blistering or crusting. Each patient underwent multiple sessions, with a mean of 23 across the series.

### Dermoscopic outcomes

The mean baseline dermoscopic pigment score was 9.0, which decreased to 2.83 at final follow-up, representing an average reduction of 6.17 points (∼68.6% improvement). One patient achieved complete dermoscopic clearance (score reduced to 0), while the remaining five patients demonstrated >70% reduction in pigment. Dermoscopy revealed a reduction of dense pigment networks, fading of dark brown to lighter brown pigmentation, blurred lesion borders, and the emergence of perifollicular clearing post-treatment.

### Clinical outcomes

All patients showed significant improvement in pigmentation (Table 2).

- One patient (Patient 2) achieved complete clearance (100% improvement) with QGS 4, PGA 4, and VAS 10.
- Three patients (Patients 3, 4, 6) demonstrated marked improvement (∼75%–80%) with QGS 4, PGA 3, and VAS 9.
- Two patients (Patients 1, 5) achieved moderate to marked improvement (∼70%) with QGS 3, PGA 3, and VAS 8.

### Patient-reported outcomes

The mean VAS satisfaction score was 8.8/10. The highest satisfaction was reported by the patient who achieved complete clearance (VAS 10/10). Patients with facial involvement consistently expressed high satisfaction. Patients also reported improvements in areas such as embarrassment, choice of clothing, and social confidence.

### Safety and adverse events

Treatment was generally well tolerated.

- Transient erythema occurred in five patients (83%).
- Post-inflammatory hypopigmentation was observed in three patients.
- Post-inflammatory hyperpigmentation was noted in one patient.
- No scarring, blistering, or long-term complications were seen.

**Figure 1.**
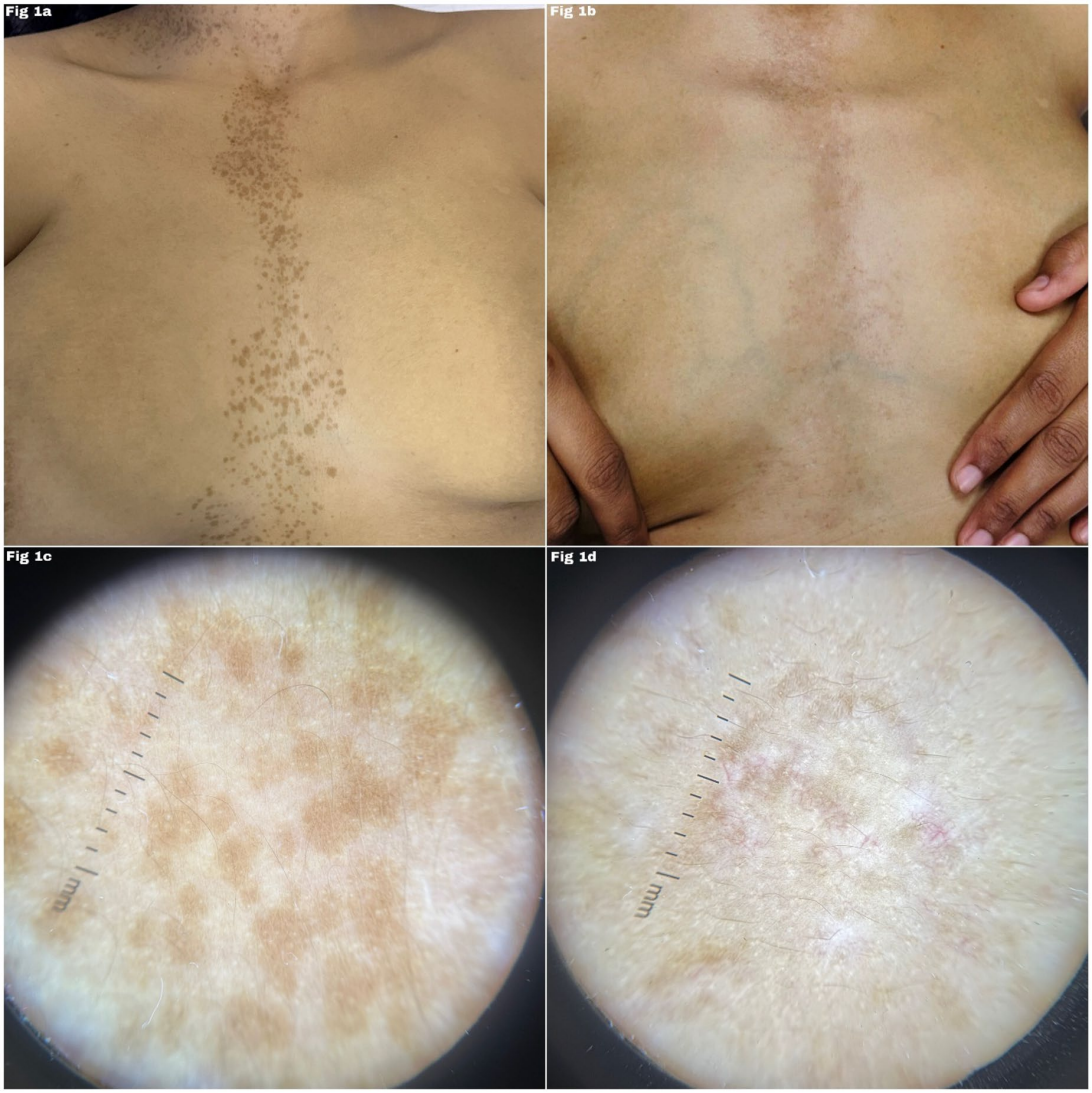
Clinical and dermoscopic images of patient 1 with partial unilateral lentiginosis showing post-inflammatory hyperpigmentation and approximately 70% clearance following Q-switched Nd:YAG (532 nm) laser treatment. (a) Baseline image showing multiple hyperpigmented macules arranged in a Blaschkoid pattern over the anterior trunk. (b) Post-treatment image demonstrating significant lightening of lesions with residual faint pigmentation. (c) Baseline dermoscopy revealing dense, irregular pigment network with dark brown globules. (d) Post-treatment dermoscopy showing fading of pigmentation, blurring of borders, and emergence of perifollicular clearing.

**Figure 2.**
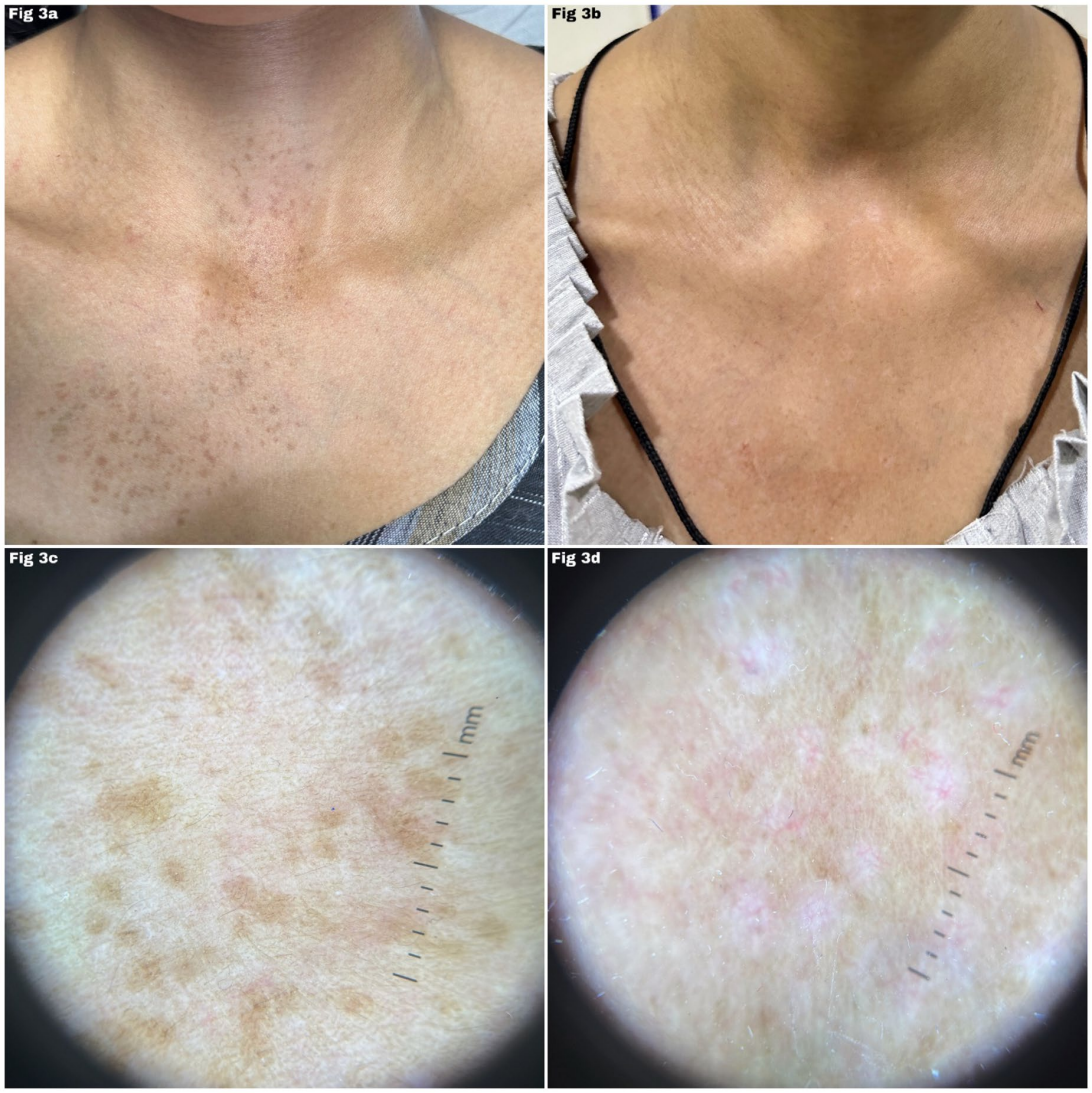
Clinical and dermoscopic images of patient 4 with partial unilateral lentiginosis showing more than 80% improvement following Q-switched Nd:YAG (532 nm) laser treatment. (a) Baseline image showing multiple light to dark brown macules arranged in a Blaschkoid pattern over the upper chest. (b) Post-treatment image demonstrating near-complete clearance of pigmentation with smooth, uniform skin tone. (c) Baseline dermoscopy showing irregular, dense pigment network with dark brown globules. (d) Post-treatment dermoscopy revealing marked reduction in pigmentation, faint residual network, and perifollicular clearing.

Representative clinical and dermoscopic images demonstrating treatment response are provided in Figures 1 and 2.

## Discussion

PUL is an uncommon pigmentary disorder for which standardized treatment guidelines are lacking. In this pilot case series, Q-switched Nd:YAG laser using the 532-nm wavelength resulted in substantial clinical and dermoscopic improvement in all treated patients, with complete clearance achieved in one case and greater than 70% improvement observed in the rest. Given the descriptive nature of the study and the small sample size, the discussion focuses on clinical trends and comparison with previously published literature rather than statistical inference.

These findings are consistent with previous reports on laser therapy for PUL, where significant pigment clearance and high patient satisfaction have been reported. Hur et al. described complete clearance in 15 patients treated with high-fluence 1064 nm Q-switched Nd:YAG laser using their “Golden Parameter Therapy,” with no recurrences during long-term follow-up [5]. Study done by Lee et al. reported favorable outcomes using low-fluence Q-switched 1064-nm Nd:YAG laser in Korean patients with facial PUL, emphasizing gradual pigment clearance with a good safety profile [9]. Similarly, Pretel et al. reported successful clearance of PUL lesions with a Q-switched alexandrite laser, further supporting the role of pigment-specific lasers in this condition [10].

While prior studies have primarily employed the 1064-nm wavelength, the 532-nm Q-switched Nd:YAG laser selectively targets epidermal melanin due to its higher absorption by melanin chromophores. As PUL is characterized predominantly by epidermal lentiginous pigmentation, the use of 532-nm wavelength efficient absorption by superficial melanin, allowing effective clearance of lentiginous macules while minimizing collateral tissue damage. The favorable outcomes observed in this series suggest that 532-nm Q-switched Nd:YAG laser can be an effective alternative in selected cases, provided treatment parameters are carefully titrated to minimize pigmentary complications [6,11].

Dermoscopy was instrumental in objectively documenting treatment response. Baseline features included dense, irregular pigment networks, heterogeneous brown pigmentation, and ill-defined borders without perifollicular clearing. Post-treatment images showed clear reduction in network density, fading of pigmentation to lighter hues, blurred lesion margins, and the emergence of perifollicular clearing. To quantify this, we employed a semi-quantitative dermoscopic pigment score (0–12) based on four reproducible parameters: pigment network density, color intensity, border definition, and perifollicular clearing. Although no validated dermoscopic score exists for PUL, structured dermoscopy scoring models have been successfully used in lentigo maligna and melanoma for diagnostic reproducibility. Adapting these principles allowed us to objectively capture improvement, and our dermoscopic scores correlated well with both clinical grading and patient satisfaction [12,13].

Recent advances in pigment laser therapy suggest that refinement of pulse characteristics may improve treatment efficiency while maintaining safety. Comparative studies have shown that picosecond-domain and photoacoustic technology–modified Q-switched Nd:YAG lasers achieve efficacy comparable to conventional Q-switched systems in pigmentary disorders. However, given the segmental nature of PUL, the applicability of these approaches remains uncertain, supporting the continued use of a cautious, low-fluence, multi-session protocol until disease-specific evidence becomes available [14,15].

A cautious, low-fluence, multi-session protocol was adopted, beginning at 0.2–0.5 J/cm^2^ and gradually escalating to a maximum of 2.5 J/cm^2^ and 200 mJ, with the clinical endpoint defined as immediate whitening and transient erythema. The relatively high number of treatment sessions in this series reflects this safety-oriented approach, particularly relevant for darker skin types that are more susceptible to post-inflammatory dyspigmentation. Gradual fluence escalation allowed consistent pigment clearance while minimizing adverse effects. Treatment was well tolerated, with transient erythema observed in most patients (83%), resolving within days. Mild pigmentary alterations were noted, including hypopigmentation in three patients (50%) and hyperpigmentation in one patient (17%), all of which were manageable and did not require treatment discontinuation. No scarring, blistering, or long-term complications were observed. Overall, these findings are consistent with the established safety profile of Q-switched lasers when carefully titrated and monitored across multiple sessions [11].

While higher-fluence or fewer-session protocols have been reported in other lentiginous conditions, their applicability in partial unilateral lentiginosis remains uncertain. Future studies with larger cohorts may explore whether moderately higher starting fluences or alternative pulse technologies can reduce the number of sessions without compromising safety.

The principal limitation of this pilot case series is the small sample size, reflecting the rarity of PUL. Consequently, findings should be interpreted as exploratory and descriptive rather than inferential. Nevertheless, pilot case series play an important role in generating preliminary evidence and informing future therapeutic approaches in rare disorders where large cohorts are difficult to assemble. Larger multicenter studies incorporating formal statistical analysis are needed to validate efficacy, optimize treatment parameters, and establish long-term outcomes. Additionally, the dermoscopic pigment score used in this study, although reproducible and clinically useful, requires validation in larger prospective cohorts.

## Conclusion

Q-switched Nd:YAG (532 nm) laser achieved complete clearance in one patient and >70% clearance in all others, delivering high patient satisfaction and demonstrating its value as a reliable treatment for partial unilateral lentiginosis. Our findings highlight this modality as a safe, effective, and reproducible therapeutic option for this cosmetically significant disorder.

## Consent

Written informed consent for publication of clinical details and images was obtained from all participants (and from parents/guardians in the case of minors). All identifying details have been removed to ensure anonymity.

## Data Availability

No new data were generated or analysed in this research.
